# Deleterious Effect of Air Pollution on Human Microbial Community and Bacterial Flora: A Short Review

**DOI:** 10.3390/ijerph192315494

**Published:** 2022-11-22

**Authors:** Nishant Gupta, Virendra Kumar Yadav, Amel Gacem, M. Al-Dossari, Krishna Kumar Yadav, N. S. Abd El-Gawaad, Nidhal Ben Khedher, Nisha Choudhary, Pankaj Kumar, Simona Cavalu

**Affiliations:** 1 Department of Medical Research & Development, River Engineering, Toy City, Ecotech-III, Greater Noida 201305, India; 2 Department of Biosciences, School of Liberal Arts and Sciences, Mody University of Science & Technology, Lakshmangarh, Sikar 332311, India; 3 Department of Physics, Faculty of Sciences, University 20 Août 1955, Skikda 21000, Algeria; 4 Research Center for Advanced Materials Science (RCAMS), King Khalid University, P.O. Box 9004, Abha 61413, Saudi Arabia; 5 Faculty of Science and Technology, Madhyanchal Professional University, Ratibad 462044, India; 6 Department of Physics, Faculty of Science, King Khalid University, Abha 62529, Saudi Arabia; 7 Department of Mechanical Engineering, College of Engineering, University of Ha’il, Ha’il 81451, Saudi Arabia; 8 Laboratory of Thermal and Energy Systems Studies, National School of Engineering of Monastir, University of Monastir, Monastir 5000, Tunisia; 9 Department of Environmental Sciences, School of Sciences, P P Savani University, Surat 394125, India; 10 Department of Environmental Science, Parul Institute of Applied Sciences, Parul University, Vadodara 391760, India; 11 Faculty of Medicine and Pharmacy, University of Oradea, P-ta 1 Decembrie 10, 410087 Oradea, Romania

**Keywords:** air pollution, human microbiota, particulate matter, autoimmune diseases, reactive oxygen species

## Abstract

A balanced microbiota composition is requisite for normal physiological functions of the human body. However, several environmental factors such as air pollutants may perturb the human microbiota composition. It is noticeable that currently around 99% of the world’s population is breathing polluted air. Air pollution’s debilitating health impacts have been studied scrupulously, including in the human gut microbiota. Nevertheless, air pollution’s impact on other microbiotas of the human body is less understood so far. In the present review, the authors have summarized and discussed recent studies’ outcomes related to air pollution-driven microbiotas’ dysbiosis (including oral, nasal, respiratory, gut, skin, and thyroid microbiotas) and its potential multi-organ health risks.

## 1. Introduction

**Figure 1:**
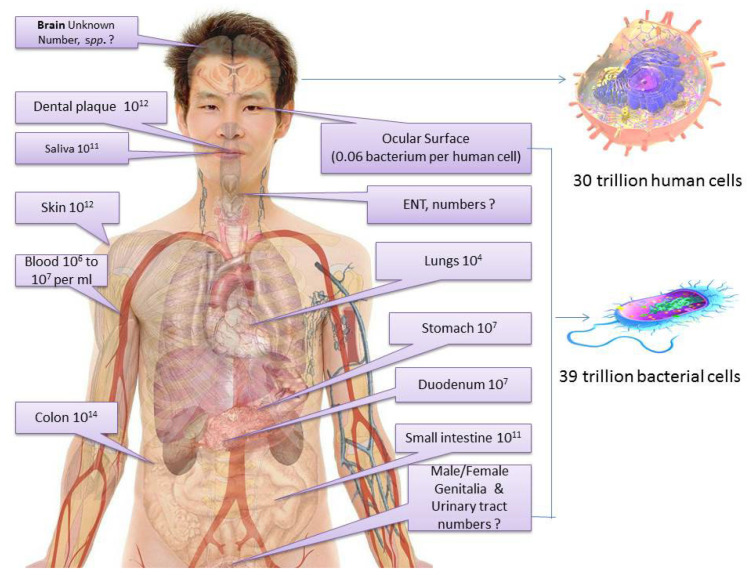
Organ-specific symbiotic bacterial cells in the human body.

The human body is the home of trillions of microorganisms including viruses, bacteria, archaea, and protists [1]. Excluding parasites and viruses, a healthy individual contains more bacterial cells than his/her own cells, approximately 30 trillion human cells and 39 trillion bacterial cells (Figure 1). However, these bacterial cells are 1000 times smaller than human cells and comprise only ~2% (1.5 kg) of a healthy adult human body mass. Usually, those bacterial cell numbers may vary from person to person [2,3,4]. Figure 1 shows organ-specific bacteria in the human body.

The numbers and arrangement of bacterial species are also different in each organ. Generally, a healthy adult human colon has the highest amount of bacteria (10^14^), followed by the skin (10^12^), dental plaque (10^12^), lower intestine (10^11^), saliva (10^11^), stomach (10^7^), duodenum (10^7^) [4], the bloodstream (10^6^ to 10^7^ per mL), eyes (0.06 bacterium per human cell) (Gomes et al., 2020), and the respiratory system (around 10^4^ microorganisms per mL) (Mathieu et al., 2018). Some studies even suggest bacterial cells are present in organs previously known as sterile, such as the brain and blood cells [5]. Pioneering microbiologist Louis Pasteur once stated that “life without bacteria would be unthinkable”. In fact, humans are considered superorganisms consisting of symbiotic microorganisms and cells [6]. Undoubtedly, a microorganism plays a significant role in human lives. In addition to dreadful infections, they also represent a crucial component of the human body; these commensal microbes in our body, collectively known as microbiota, are required for vital functions [7]. In particular, bacteria are a major part of the human microbiota.

These beneficial, neutral, and sometimes opportunistic microbes within the human body are known as normal microbial flora. The human body usually retains these microbes from birth. However, a few studies detected microbial cells in womb tissues as well. The normal microbiome of the human body influences physiology, anatomy, and morbidity as well as susceptibility to pathogenic germs. However, there are many factors that alter the normal microflora compositions and contribute to turning them into opportunistic pathogens [8].

Recent studies suggested air pollution’s role in symbiotic microbial dysbiosis (imbalance). Even short-term exposure to air pollutants showed strong effects against microbial interactions [9].

Air pollution may also exacerbate or facilitate pathogens’ infections such as tuberculosis, meningitis [10], and COVID-19 mortality [11,12]. Recent studies have highlighted air pollution’s role in previously less-studied gastrointestinal diseases such as increased peptic ulcer hospitalization due to PM_10_, O_3_, and NO_2_ air pollutants [13].

The debilitating impact of air pollution on human health is widely studied. Air pollution exposure can increase risk to maternal health and neonatal health and susceptibility to respiratory diseases, neurological disorders, cognitive ailments, cancers, and diabetes. Air pollutants enter the human body through the respiratory pathway and dissolve in the bloodstream, which may trigger inflammation, oxidative stress, immunosuppression, and mutagenicity in cells, affecting the respiratory system and other internal organs and ultimately causing diseases [14].

Undoubtedly, air pollution is a serious environmental and health concern. According to the WHO’s recent update, air pollution has affected almost all the global population; currently, 99% of people breathe air that exceeds the WHO clean air quality limits. However, the proportion and exposure of air pollution are highest among low- to middle-income nations, especially in Asian countries [15]. According to the United States Environmental Protection Agency (US EPA), common air pollutants, such as particulate pollutants (PM_2.5_, PM_10_), sulphur dioxide (SO_2_), carbon monoxide (CO), lead (Pb), ozone (O_3_), and nitrogen dioxide (NO_2_), are the most harmful pollutants to environmental and human health [16].

There is limited but growing evidence suggesting that exposure to environmental pollutants may lead to the dysbiosis of the human microbiota including the respiratory tract, female reproductive system, and gut. Dysbiosis of the microbiota may facilitate the overgrowth of pathogens and disrupt beneficial metabolite production, resulting in numerous health issues [17].

Most of the existing research and systematic reviews have focused on air pollution’s impact on particular types of human microbiotas such as the gut microbiota, which is considered most important for overall health. The gut microbiota was found to be extremely sensitive to drugs, diet, and even environmental pollutants [18]. However, the direct impact of air pollution on the gut seems to be underestimated [19]. Similarly, very few or negligible studies have focused on the relationship between air pollution and other microbiotas.

**Scheme 1:**
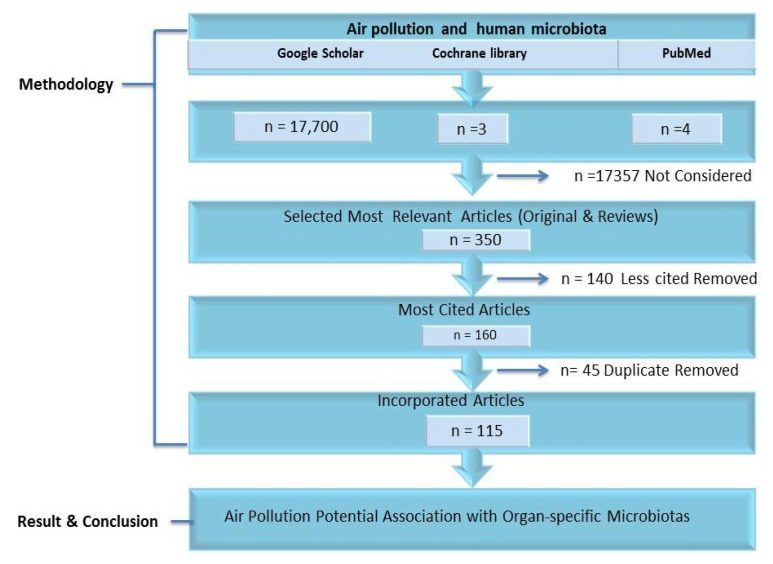
Methodology of this study.

For this review article, the authors evaluated and discussed the existing scientific literature on air pollution’s impact on different microbiotas and potential health outcomes in the human body. The information was summarized on the basis of currently available related research and reviews. The investigators emphasized the effect of pollutants on the normal microflora of human beings. The following flow chart (Scheme 1) explains the methodology of this study.

## 2. Methodology

In this article, Google Scholar, Cochrane and PubMed databases were mainly used to fetch scientific data. Common filters such as the English language, reviews, and publication years between 2010 and June 2022 were applied. A search on the keywords human microbiota and air pollution was used during the scientific literature searches in selected databases. Additional relevant keywords such as human microbiota, the human microbiome, air pollution, gut microbiota, air pollution, respiratory microbiota, and so on were also used to fulfil the required gap. Recent studies were mostly prioritized. A total of 17,700 articles (7750 reviews) appeared when searching for human microbiota and air pollution. Out of those, 350 articles were found to be relevant to this review. After removing irrelevant and duplicate articles, only 160 articles were considered for the final review. Research data inclusion criteria were based on the motive of this review study. We selected only those articles that fulfilled our review article theme and objective. Irrelevant, repeated articles were excluded. Further duplicates and relevant studies’ abstracts, conclusions, and results were resolved (read and edited) by the reviewers. Finally, articles (reviews and originals) were used for referencing and incorporating.

## 3. Air Pollutants and Dysbiosis of Human Microbiota

Alterations in the microbiota can be due to exposure to various environmental factors [20]. Air pollutants such as particulate matter (PM_0.1_, PM_2.5_, PM_10_), O_3,_ and polycyclic aromatic hydrocarbon (PAH) inhalation may alter the human microbiota by various pathways, depending on the type of exposure. In particular, there are several courses for air pollutants and gut microbiota interactions. For instance, the gastrointestinal tract may have exposure to air pollutants via inhalation and ingestion. Usually, large particulate pollutants are deposited in the upper airway such as the trachea; but the finer pollutants, such as PM_2.5_, reach into the lung’s alveolar space, further moved and hidden by alveolar macrophages [21,22].

Air pollutants are associated with dysbiosis of the human microbiota, particularly the gastrointestinal microbiota. Air pollutant polycyclic aromatic hydrocarbons (PAH) may alter the commensal as well as environmental microbial communities. Air pollutant PAH can affect the purine pyrimidine metabolites’ signaling pathways and lipid metabolism [22]. An animal (broilers) study showed ammonia inhalation can also alter tracheal and ileal microbiotas by the TLR4 signaling pathway [23].

Studies showed that high concentrations of inhaled particulate matter are transported through the mucociliary pathway and that the pollutant is rapidly cleared from the lung’s alveolar cells but further transported to the intestine. Ingested particulate matter triggered gastrointestinal inflammation-induced dysbiosis of gut microbes [24]. Gut microbiota dysbiosis causes several health issues including mental health via the gut-brain axis. Gut microbes can influence the central nervous system via the vagus nerve [25,26]. Particulate matter (PM) and heavy metal air pollutants are exposed to the gut via inhalation or ingestion. PMs cause gut dysbiosis, which further disturbs or alters the composition of gut microbiota-associated regulatory metabolites such as short-chain fatty acid (SCFA), which is required for brain-gut neurotransmitter signals [27].

Short-chain fatty acid (SCFA) is the predominant anion and is usually produced by the commensal gut bacterial fermentation of undigested carbohydrates. Interestingly, up to 95% of SCFA is absorbed by colon cells (colonocytes) as an energy substrate. Colonocytes usually derive their energy (up to 70%) from SCFA’s oxidation, which provides nearly 10% of the daily calories required for the human body.

**Figure 2:**
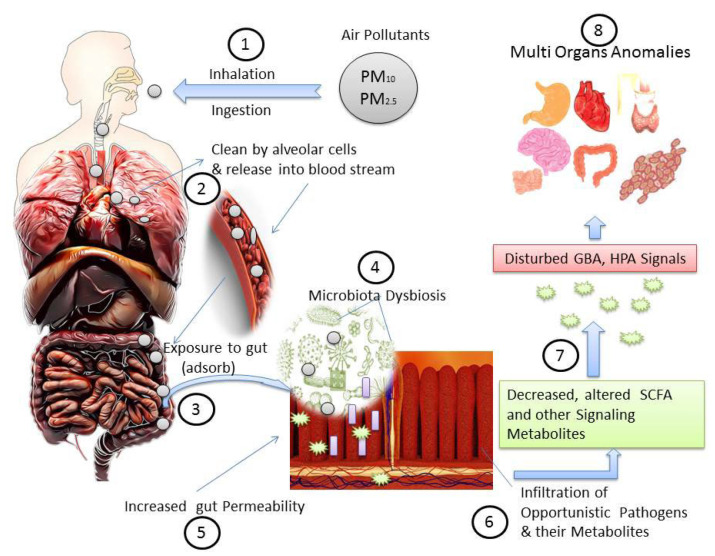
A possible mechanism of air pollution associated gut microbiota dysbiosis and health issues: (1) air pollutant entry; (2) respiratory system response; (3) reached to gastrointestinal tract and adsorbed; (4) dysbiosis and alteration of commensal gut microbiota; (5) altered microbial cells and their metabolites increased gut permeability and susceptibility to pathogens; (6) infiltration of harmful metabolites and pathogens decrease gut microbiota-dependent metabolites such as short-chain fatty acid (SCFA); (7) disturbed gut-brain axis (GBA) and hypothalamus–pituitary–adrenal axis (HPA); (8) altered signaling metabolites influence several organs’ functionalities.

Most studies focused on gut microbiota dysbiosis, due to its role in several diseases and since the gut microbiota is associated with various ailments and may influence host cognitive function and behavior by communicating altered signals to the hypothalamus–pituitary–adrenal (HPA) axis [20]. Figure 2 shows a possible mechanism of air pollution associated with dysbiosis of gut microbiota and health issues.

Another proposed mechanism explains how ingested pollutants enter and are further adsorbed into the intestine from the bloodstream. These pollutants are also oxidized into the liver, form conjugates such as glutathione, and are excreted into the intestine again. The gut microbiota interferes with normal glutathione secretion; gut microbes additionally metabolize those pollutants, which disturb their composition as well, which could be responsible for the inactivation or re-activation of the specific metabolites or compounds responsible for various associated health conditions including cancer [28]. Certain commensal bacterial species such as *Escherichia coli* also turn invasive and cause metabolic endotoxemia, which increases the permeability of the gut wall, decreases mucus thickness, and allows pathogenic invasive bacterial species to infiltrate [29].

Studies have suggested air pollution’s negative impact on human microbiome development, which is associated with serious health outcomes including obesity, gastric ailments, and autism. Up to this time, air pollution garnered less attention despite the potential risk of negative alterations [8,30].

Several studies have explained gut microbiota dysbiosis; however, it is a multifactorially dependent, complex event to understand clearly, so far. More comprehensive research is required to understand air pollutant exposure associated with microbiota dysbiosis, especially to explain the other microbiotas’ dysbiosis of the human body.

Air pollution’s impact on the gastric microbiota has been studied extensively. Up to this time, its impact on other organ-specific microbiotas was studied sparsely. In this mini-review, we discussed air pollution’s potential impact on the microbiotas that belong to different organs of the human body.

## 4. Air Pollution Impact on Nasal Microbiota

The human nasal tract is the home of diverse bacterial communities. Nasal microbiota plays a pivotal role in personal and public health as well. Human nostrils are colonized by various microbes just after birth. Usually, pediatric and adult nasal microbiotas are distinguished from each other. Pediatric nasal microbiota usually belongs to the Gram-negative bacteria Haemophilus and Moraxella and Gram-positive bacteria Streptococcus, Staphylococcus, Dolosigranulum, and Corynebacterium. In the case of adults, the nasal microbiota is mostly Gram-positive (Firmicutes and Actinobacteria). Despite ample exposure to oxygen, the most frequent nasal bacterial species are either facultative anaerobes or aerotolerant. There are lesser-known factors that influence and alter nasal microbiota; age, season, and climate may also alter the nasal microbiota [31]. Additionally, air pollution, even low exposure, may influence the nasal microbiota during the first year of life; especially, exposure to air pollutants PM_2.5_ and NO_2_ is associated with nasal microbiota alteration, respiratory infections, and asthma development during early life [32,33].

Nasal microbiota is different from the upper respiratory tract (URT). The upper respiratory tract has constant airflow, which prevents the spread of pathogens to the lower respiratory tract. Usually, the nasal microbiota remains almost unchanged in adulthood. A few studies suggested that the nasal microbiota has been significantly associated with the central nervous system and immune system. Dysbiosis in the nasal microbiota may lead to inflammatory diseases and pathological invasion of the nasal cavity, which may damage the olfactory system [34].

The altered composition of beneficial bacteria residing in the nasal sinus cavity was found to be associated with Parkinson’s disease [35]. Growing evidence suggests that particulate air pollutants (PM_2.5_, PM_10_) may alter the nasal bacterial community [36,37].

## 5. Air Pollution and Oral Microbiota Dysbiosis

A balanced, normal composition of oral microbiota is required for the oral and probably overall health of the host. Being a compatible environment, the oral cavity facilitates the growth of many distinct commensal as well as harmful microbes. In recent years, the importance of the oral microbiome has also increased in dental medicine. The oral cavity is susceptible to constant environmental factors including host eating habits, saliva secretion, mastication, and exposure to outside microbes [38,39]. Air pollutants such as ultrafine particles (PM_0.1_) can affect the oral microflora and promote inflammation in the oral cavity of children [40].

In 2010, research highlighted the importance of the oral microbiome for the first time. Most recently, oral microbiota dysbiosis has emerged as one of the contributing factors to diseases such as schizophrenia and osteoporosis. Until now, probiotics were found to be useful for the restoration of oral dysbiosis [41]. Similarly, several molecules from saliva form a specific film to minimize dysbiosis; therefore, the process of dysbiosis may increase if the amount of saliva decreases [42].

Oral microbiota may influence other distant organs. Altered oral microbiota can disseminate to other organs via ingestion and systemic circulation. Recent findings indicate an association between altered oral microbiota and liver and pancreatic diseases [43], oral cancer [44], and Alzheimer’s disease [45]. A recent metagenomic analysis revealed that oropharyngeal microbiota dysbiosis was linked with COVID-19 severity [46]. In addition, oral and gut microbiotas’ crosstalk may increase the complication of oral dysbiosis to other organs [47].

## 6. Air Pollution Impact on Pharyngeal Microbiota

The pharyngeal microbial communities are important in the airflow cavity to stimulate the immune system and prevent unwanted pathogenesis of airborne germs. These local microbes provide a defense line against newly emerging pathogens. The most common pharyngeal microbiota belongs to bacterial genera such as *Campylobacter*, *Capnocytophaga*, *Haemophilus*, *Neisseria*, *Streptococcus*, *Prevotella*, and *Veillonella*. To retain proper health, a balance of common microbiota is necessary for all. However, the epithelial microbial community in the pharynx can be damaged by factors such as air pollutants, smoking, and infections [48].

It has been reported that even a few days of exposure to particulate air pollutants may alter the pharyngeal microbiota composition, which can increase the chance of respiratory infections [49]. A similar study in China concluded that air pollution can lead to oropharyngeal microbiota dysbiosis. The microbial population may differ as per the air pollutants’ concentration. For instance, in most polluted regions, bacteria belonging to *Fusobacteria* and *Bacteroidetes* were significantly lower while others, such as Proteobacteria, *Firmicutes*, and *Actinobacteria*, were found to be higher in participants [50].

A recent experiment analyzed the nature and characteristics of pharyngeal bacteria among chronic pharyngitis patients using 16S rDNA-based detection technology. The study concluded that the community and quantity of symbiotic pharyngeal bacteria decreased while the number of opportunistic bacteria increased in chronic pharyngitis patients [51]. Pharyngeal microbiota’s abundance and specificity have also been associated with age-related macular degeneration [52].

The pharynx microbiota’s potential role between gut and lung crosstalk is being investigated. A clinical study on neonates’ pharynx and intestine microbiotas showed a significant difference between intestinal and pharynx microbial species and composition. However, some species such as Streptococcus were common in both [53].

## 7. Air Pollution and Altered Respiratory Microbiota

The respiratory system is a complex system that facilitates oxygen and carbon dioxide exchange. Nostrils of the lungs and alveoli of the human respiratory system are inhabited by specific bacteria, which are known as the common microbiota of the respiratory tract and work as a gatekeeper to prevent the colonization of pathogens. Respiratory microbiota also maintains respiratory physiology and immunity. Despite the respiratory microbiota’s important role in mediating human health response to inhaled irritants, the respiratory microbiome and inhaled pollutants’ association have remained less explored [54].

Healthy lung microbiota usually belongs to *Firmicutes* and *Bacteroidetes*. Alteration of the respiratory microbiome is associated with inflammation and chronic lung diseases. Polluted air introduces a mixture of air pollutants such as PM_2.5_, PM_10_, O_3_, NO_2_, CO, SO_2_, polycyclic aromatic hydrocarbons, and pathogens into the respiratory system. The effect of polluted air is bidirectional, as air pollutants such as PM_2.5_ penetrate deeply into lung cells and damage epithelial integrity, which further facilitates the entrance of harmful microbes and toxic metabolites into the epithelial layer and triggers systemic immune activation, which causes alteration or dysbiosis of the lung microbiota. Air pollutants also provoke the production of reactive oxygen species (ROS), which directly kills and reduces the resident microbiota [55].

A study on 40 children showed that exposure to automobile air pollution in childhood and adolescence may be associated with significant changes in the lower respiratory microbiota, which may lead to asthma. Alteration and dysbiosis of the respiratory microbiota can increase the chance of lower and upper respiratory tract infections. In particular, lower respiratory infection such as pneumonia is responsible for considerable morbidity and mortality among children under 5 years globally [56]. A cross-sectional study on healthy adults in Malawi showed that higher exposure to particulate air pollutants caused an abundance of the potentially pathogenic bacteria Streptococcus and Neisseria in their lungs [57]. Therefore, air pollution can affect the respiratory microbiota and induce dysbiosis, which is associated with several respiratory diseases [58].

## 8. Air Pollution and Skin Flora

Certainly, the skin is the largest organ of the human body and is colonized by diverse microbial species, mostly harmless or even beneficial. However, some microbes are potential components of many skin disorders as well [59].

The skin microbiome is said to be an integral part of the skin protective layer that can affect skin health by modulating the host’s immune system. Therefore, dysbiosis or alteration in skin microbiota may be responsible for poor skin health and various diseases. A study conducted in Shanghai (China) showed that air pollutants from heavy traffic can weaken the skin’s physical and antioxidant barrier properties. By analyzing facial microbiome sequencing, the same study suggested that air pollution had an apparent effect on the fragility of the skin microbial network, thus damaging skin health, and a smoking lifestyle further amplified the damaging effects of air pollution on the microbiome and the skin [60,61].

Skin flora may be affected by other common and more frequent air pollutants such as particulate matter PM_2.5_. Resident cutaneous microflora helps to maintain homeostasis and prevents outside harmful microbial infection, but ambient air pollution is found strongly associated with the alteration of skin microbiota. Air pollutant O_3_ exposure is bactericidal and can reduce the resident skin microflora by 50% [62]. Air pollutants such as polycyclic aromatic hydrocarbons (PAHs) [63] and long exposure can also change the composition and capacities of the cutaneous microbiota [64].

On the other hand, the air pollution effect on the skin is not studied well despite the skin being one of the most common and easiest targets for air pollutants. Air pollutants may penetrate the skin more deeply by transcutaneous and systemic routes; this causes several skin problems including abnormal skin aging [65].

## 9. Air Pollution, Gut Microbiota Dysbiosis, and Associated Diseases

The gut microbiota is pivotal for health; in fact, the gut microbiota can determine one’s health status. The gut microbiota partly depends on interactions with the host genotype and the environment. Numerous studies have described that a change in gut microbiota composition (dysbiosis) is associated with several health issues in the host [66]. The gut microbiota is probably the most studied microbiota over the other organ microbiotas in the human body. Gut microbiota’s role in gastrointestinal health, as well as diseases, has been described widely [67].

The human gut represents a dense microbial milieu; approximately 10^14^ bacteria and archaea belong to 1000 species [68]. As compared to the other symbiotic microbes, human gut microbiota represents a separate niche of symbiotic microbes. The human gut is the hidden garden of trillions of diverse symbionts. Even the human stomach, previously thought to be sterile, contains a significant number of symbiotic microbes as well. A healthy human stomach is normally inhabited by five major phyla of bacteria, viz., *Firmicutes*, *Bacteroidetes*, *Actinobacteria*, *Fusobacteria*, and *Proteobacteria*, and five bacterial genera: *Prevotella*, *Streptococcus*, *Veillonella*, *Rothia*, and *Haemophilus* [69].

The symbiotic association between the host and gut microbiota is a complex network system. It is believed that the crosstalk between the host and gut microbes is mediated by microbial metabolites, which act as signaling molecules and regulate the host neuro–immune–inflammatory axes that link the gut with other organ systems of the host [70]. The gut microbiota in human health is a rapidly growing topic in scientific and medical knowledge. New research is continuously explaining gut microbes and human health associations [71].

Growing evidential studies show dysbiosis in the host gut microbiota may be responsible for many systemic diseases (Table 1). Numerous diseases, ranging from liver diseases to neuropsychiatric diseases, are associated with gut microbiota dysbiosis. Liver bile secretion also regulates the composition of the gut microbiota. Similarly, in turn, intestinal microbes help to induce and regulate the synthesis of bile acids by the FXR/FGF-19 axis and secrete several incretins for lipid and glucose metabolism into the bloodstream. Alteration or dysbiosis in the gut microbiota significantly affects the quality and quantity of bacterial and host metabolites that reach the liver and modulate molecular pathways [72,73].

Gut microbiota dysbiosis is defined as the imbalance of gut microbiota associated with an unhealthy outcome. Dysbiosis often involves the loss of healthy microbial species and the expansion of unhealthy or pathogenic microbial species. Various factors can prompt the dysbiosis of gut microbiota, including air pollution, lifestyle modification, cigarette smoking, nicotine-based products, unhealthy diet, and lack exercise [74,75].

Air pollution causes several gastric problems by directly affecting the gut microbiota. The gut microbiota has also been found to be associated with neurological and cardiovascular systems dysfunction as well [96].

## 10. Potential Joint Role of Air Pollution and Microbial Dysbiosis in Diseases’ Burdens

Air pollution-associated dysbiosis may contribute to the worldwide burden of some diseases such as cancer, IBS, and thyroid dysfunction.

### 10.1. Irritable Bowel Syndrome and Inflammatory Bowel Diseases

IBS and IBD burdens are rising immensely and globally. An estimated 3.9 million females and nearly 3 million males live with IBD worldwide [97], while IBS also remained a critical health issue for 10–20% of the population worldwide. IBS etiology is multi-factorial and may be associated with factors such as immune disorders, genetics, infections, psychological factors, and alterations in microbiota. However, which of these factors is the main trigger for the onset of IBS is not completely known. Although the role of environmental pollution to IBS association has not been fully understood, available studies suggest that it is one of the key factors in IBS pathophysiology [98]. However, gut microbiota dysbiosis is strongly associated with IBS [99,100].

Similarly, inflammatory bowel disease has remained a challenging health problem so far [101]. IBD manifests as ulcerative colitis or Crohn’s disease. The link between IBD and air pollution is not clearly understood. Some studies indicated no direct link between IBD and air pollution; but air pollutants such as SO_2_ and NO_2_ were found to be associated with the early onset of ulcerative colitis and Crohn’s disease [102,103], while other studies suggested air pollution can increase the risk of IBD [104,105]. In particular, ingested air pollutants (particulate matter) could accelerate inflammatory bowel disease [24].

Since the gut microbiota is dynamic in nature and can influence the physiology and metabolism of the human body, disturbances in the gut microbiota not only cause gastrointestinal ailments but also increase the risk of other diseases such as cardiovascular diseases [94]. Air pollution associated with microbial dysbiosis may also contribute to the burden of IBS and IBD.

### 10.2. Disturbed Thyroid Functions

In 2012, an estimated 200 million individuals suffered from thyroid disorders worldwide [106]. A healthy gut microbiota is essential for normal thyroid functions. It was reported that thyroid diseases such as Graves’ disease, Hashimoto’s thyroiditis, and the most common autoimmune thyroid diseases are often associated with gut microbes’ dysbiosis. The gut microbiota plays a significant role in thyroid functions and maintains the thyroid–gut axis. Healthy gut microbes regulate micronutrients such as iron, iodine, copper, zinc, and selenium essential for thyroid hormone synthesis [107]. Similarly, a meta-analysis showed the alteration of the gut microbiota composition is significantly associated with autoimmune thyroid disease [108].

An animal study examined the air pollution particulate matter (PM_2.5_) impact on intestinal microbiota and thyroid functions. The findings showed a significant relationship between pollutant PM_2.5_-derived intestinal microbes’ dysbiosis and associated thyroid hormonal imbalance [95].

### 10.3. Microbial Dysbiosis and Carcinogenesis

In 2020, an estimated 19.3 million new cancer cases and 10.0 million cancer deaths were reported globally [109]. Growing evidence shows that the balanced human microbiota has a great deal of health-promoting and cell proliferation-maintaining potential. However, an imbalance in microbiota invites several pathogens that further cause inflammation and produce genotoxins and several carcinogenic metabolites [110]. In addition, disturbing organ-specific microbiota may also promote carcinogenesis; however, the complete mechanism is less understood so far. Changes in commensal respiratory bacterial species’ composition may be related to lung cancer [60]. Similarly, colorectal carcinogenesis is also associated with gut microbiota perturbance [111]. Diverse bacterial communities are present in the normal breast microbiota; altered microbial composition can increase breast cancer risk. Changes in the gut microbiota may affect several microbial metabolite productions, such as secondary bile acids (SBAs), mainly synthesized by intestinal bacteria; irregular production may increase the risk of the colon as well as liver cancer [112,113].

Mucosal tissue samples from a recently diagnosed bladder cancer patient showed that harmful chemical exposure from the environment may cause bladder microbiota dysbiosis, which is associated with increased bladder cancer incidence [114].

## 11. Rebalance and Prevention of Microbial Dysbiosis

Altered gut microbiota is usually managed by probiotic and prebiotic administration, together known as synbiotics. These therapies replace harmful microbes with beneficial ones. Other, similar therapeutic strategies such as phage therapy and fecal transplantation are still limited and being investigated [115]. Since microbiota dysbiosis is associated with diverse health issues, by managing gut microbes’ healthy composition the risk of associated diseases can be minimized. Personalized microbiome therapy and probiotic intervention can also improve the gut microbe composition and enhance immunomodulatory functions of the gut. Similarly, postbiotics are those metabolites that are secreted by the gut microbiota and responsible for various biological activities. Short-chain fatty acids are the most common and known example of postbiotics. In some cases, postbiotics were found to be effective and safer as compared to viable bacteria [116]. Air pollution-associated microbial dysbiosis treatment and prevention literature are scarce. However, common preventive measures such as indoor air cleaning technologies including HEPA filter-based air cleaners, air ionizers, and air purifying plants may be helpful to reduce exposure to dysbiosis-causing air pollutants [117].

## 12. Conclusions

It is now well accepted that the human gastrointestinal tract (GIT) gathers the most diverse and dense microbiota of the human body, which, in turn, plays fundamental roles in gut homeostasis. Air pollutants are inhaled into the lungs. The smaller particles can reach the alveolar space where they can be phagocytosed by alveolar macrophages and consequently transported to the oropharynx and into the GIT.

Air pollutants may alter the composition of the normal microflora of the human body. Studies have indicated air pollution’s role in gut microbiota dysbiosis and its negative outcomes. Since the gut microbiota acts as the control room to regulate several systemic functions or acts, it is associated with the gut-brain axis.

**Figure 3:**
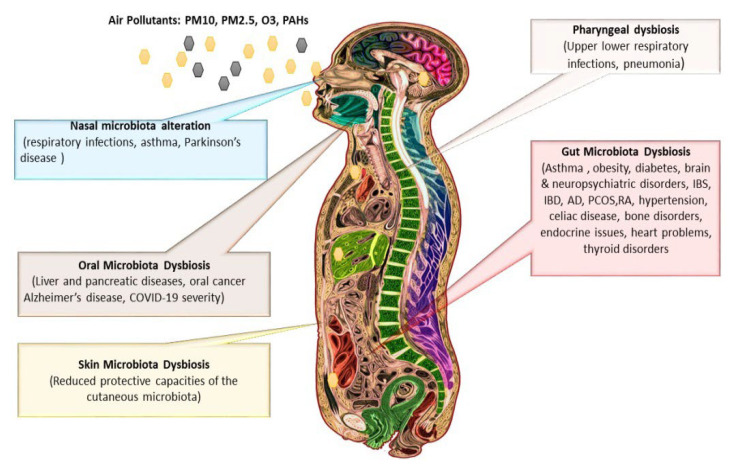
Air pollution-associated microbial dysbiosis in different organs and potentially associated health risks.

Disturbance in the gut microbiota may be responsible for several systemic diseases and multiple organ functionalities, including liver and neuropsychiatric diseases. The mechanism of gut microbiota dysbiosis explains how altered microbial cells and their metabolites may increase the gut permeability and facilitate the growth of harmful pathogens and the translocation of their metabolites, such as how short-chain fatty acid perturbs the signaling of the gut-brain axis (GBA) and hypothalamus–pituitary–adrenal axis (HPA) and may cause multiple organs’ dysfunctions. Studies show organ-specific microbial dysbiosis’s effect on human health. However, there is limited evidence of how microbiotas of different parts of the human body are affected by air pollutants. In this review, we addressed the potential role between air pollutants and other microbiotas of the human body including respiratory, oral, nasal, gut, and skin (Figure 3). However, more extensive literature and clinical studies are needed to understand the complex role of air pollutants in different organ microbiotas’ dysbiosis and the associated health risks.

## Figures and Tables

**Table 1:** Some health conditions are associated with gut microbiota dysbiosis.

Diseases Associated with Abnormal Gut Microbiota	Findings	References
Asthma in children	A little wave of air pollution may trigger asthma by impacting intestinal bacteria.	[76]
Weight gain and obesity	An imbalance in gut microbiota, especially *Bacteroidetes* and *Firmicutes*, is associated with obesity.	[77]
Diabetes, insulin resistance, inflammation	The imbalanced gut microbiome causes abnormal production of metabolites, inflammatory reactions, glucose metabolism alteration, and even insulin resistance.	[78]
Type 1 diabetes	Gut bacterial dysbiosis is highly associated with Type 1 diabetes.	[79]
Brain disorders	Impairment of gut microbiota may cause the development of autism and mood disorders.	[80]
Depression	Gut microbiota may be associated with depression.	[81]
Neuropsychiatric disorders	A link exists between gut microbiota and Alzheimer’s disease, major depressive disorder, and schizophrenia.	[82]
Irritable bowel syndrome (IBS)	Bacterial gut disturbances have been linked to the development and severity of IBS.	[83]
Bone disorders	Microbiota dysbiosis has been linked with multiple bone disorders.	[84]
Endocrine abnormalities	Gut microbiota has emerged as an important factor in endocrine system diseases.	[67]
Aneurysms	Gut microbiota contributes to the pathophysiology of aneurysms by modulating inflammation.	[85]
Sleep quality	A novel association exists between sleep health and gut microbiome diversity.	[86]
Adverse effects on organ transplantation	Disturbances in gut microbiota populations are associated with a number of adverse outcomes.	[87]
Inflammatory bowel disease (IBD)	The gut microbe’s dysbiosis may play a pivotal role in the pathogenesis of IBD.	[88]
Autoimmune diseases (AD)	Alterations in gut microbiota composition are associated with multiple autoimmune diseases.	[89]
Polycystic ovary syndrome (PCOS)	Dysbiosis of gut microbiota has been found to be associated with the pathogenesis of PCOS.	[90]
Rheumatoid arthritis (RA)	Change in gut microbiota has been associated with the pathogenesis of RA.	[91]
High-altitude (HA) sickness	Gut microbiota may contribute to variability in host responses to HA.	[92]
Hypertension	Variation in gut microbial parameters was likely associated with Chinese patients with hypertension.	[93]
Celiac disease	Dysbiosis of gut microbiota may lead to celiac disease.	[66]
Cardiovascular diseases	Disturbance in gut microbiota may lead to cardiovascular diseases.	[94]
Thyroid dysfunction	Animal study showed PM_2.5_ exposure disturbed the vital gut–thyroid axis and metabolic pathways related to thyroid thyrotoxicosis.	[95]

## Data Availability

Not applicable.
